# The Association between E326K of *GBA* and the Risk of Parkinson's Disease

**DOI:** 10.1155/2018/1048084

**Published:** 2018-04-01

**Authors:** Yongpan Huang, Langmei Deng, Yanjun Zhong, Minhan Yi

**Affiliations:** ^1^Information Security and Big Data Research Institute, Central South University, Changsha, Hunan, China; ^2^Department of Pharmacology, Institute of Chinese Medicine, Hunan Academy of Chinese Medicine, Changsha, Hunan, China; ^3^Department of Emergency, The Third Xiangya Hospital and School of Life Sciences, Central South University, Changsha, Hunan, China; ^4^ICU Centre, The Second Xiangya Hospital, Central South University, Changsha, Hunan, China; ^5^Department of Ecology and Evolutionary Biology, University of Michigan, Ann Arbor, MI, USA

## Abstract

It is reported that both the homozygous and heterozygous states of GBA mutations which are the causes of Gaucher disease (GD) are linked to the risk of PD. However, the GBA variant p.E326K (c.1093G > A, rs2230288), which does not result in GD in homozygous carriers, has triggered debate among experts studying Parkinson's disease (PD). In order to determine if the E326K variant of GBA is associated with the risk of PD, a standard meta-analysis was conducted by searching and screening publications, data extraction, and statistical analysis. Finally, a total of 15 publications, containing 5,908 PD patients and 5,605 controls, were included in this analysis. The pooled OR of the E326K genotype analysis was 1.99 (95% CI: 1.57–2.51). The minor allele frequencies of E326K for PD patients and controls were 1.67% and 1.03%, respectively. The pooled OR for the minor allele A was 1.99 (95% CI: 1.58–2.50). According to the subgroup analysis, we found that the significant differences between PD patients and controls for both genotype and allele of E326K also exist in Asians and Caucasians, respectively. In this study, we found that E326K of GBA is associated with the risk of PD in total populations, Asians, and Caucasians, respectively. Further studies are needed to clarify the role of GBA in the pathogenesis of PD.

## 1. Introduction

Parkinson's disease (PD) is a common neurodegenerative disorder, with a prevalence of 1% in a population greater than 60 years old [1]. Though the etiology of PD remains unclear, it is understood that genetic, environmental, and aging factors play a role in the occurrence of PD [2].

Pathogenetic mutations in the glucocerebrosidase gene (*GBA*), encoding lysosomal enzyme glucocerebrosidase (GCase), are the cause of Gaucher disease (GD) [3]. It is reported that both the homozygous and heterozygous states of these mutations are linked to the risk of PD [4]. Moreover, PD patients with *GBA* mutations are more likely to have an early age onset, initial bradykinesia, and a family history of dementia [5]. Furthermore, screening of *GBA* in PD patients has found other potentially related variants, including p.E326K (c.1093G > A, rs2230288). E326K was named in accordance with the tradition nomenclature, which was excluded the first 39-residue signal peptide of *GBA* protein and is widely used in this field. In fact, it is the same as p.E365K, which was the recommendation of HGVS nomenclature. In the studies conducted by Ziegler's team of researchers [6] and Duran and his colleagues [7], the E326K variant increased the risk of PD. This association, however, was not observed in other studies [4, 8]. In order to evaluate the association of E326K with risk of PD, we performed a meta-analysis to clarify the general findings of large-scale results.

## 2. Methods

### 2.1. Literature Search

Databases that included PubMed, Embase, and Web of Knowledge were utilized up to July 30, 2017, with the following key words: (“parkinson^∗^” or “PD”) and (“*GBA*” or “glucocerebrosidase”). EndNote was used to manage and organize all searched publications.

### 2.2. Inclusion and Exclusion Criteria

All eligible studies had to fulfill the following inclusion criteria: (1) case-control design; (2) all PD cases diagnosed accurately according to reported criteria; (3) none of the controls had PD or a neurological disease; and (4) the genotype results of E326K were named by traditional nomenclatures, and HGVS nomenclatures were converted to traditional for both case and control groups. The exclusion criteria were as follows: (1) duplicate articles found in different databases; (2) different manuscripts using an overlapping study population; (3) genetic screening results lacking sufficient data to calculate the odds ratio (OR) and 95% confidence interval (CI); and (4) reviews. Overlapping articles from different databases were excluded with the help of electronic and manual checking. Two researchers performed the search independently. In the case of opposing opinions or decisions, a third researcher was asked to arbitrate.

### 2.3. Data Extraction

Two authors independently performed extraction of the following information from studies meeting inclusion criteria: publication date (year), first author, country of origin, sequencing method, total numbers, and responsive number of E326K genotypes (GG/GA/AA) and alleles (G/A) in PD patients and controls. If there were conflicts, a third party was asked to make a final decision. In terms of the assessment of a publication's quality, the Newcastle–Ottawa Scale (NOS) [9] was used.

### 2.4. Statistical Analysis

All statistical analyses were conducted in RevMan 5.3 software. Pooled odds ratio (OR) and 95% confidence interval (CI) were applied to measure the strength of associations between E326K and PD. Heterogeneity among all studies was calculated with a standard Q test. A fixed model (FM) was applied when the heterogeneity was not significant (*P* > 0.1; *I*^2^ ≤ 50%), or a random model (RM) was used. Publication bias was measured through funnel plot analysis. Sensitivity analysis was conducted by removing each individual publication from the pool of all the included studies and then reanalyzed the remaining pool to measure the stability of the results.

## 3. Results

**Figure 1:**
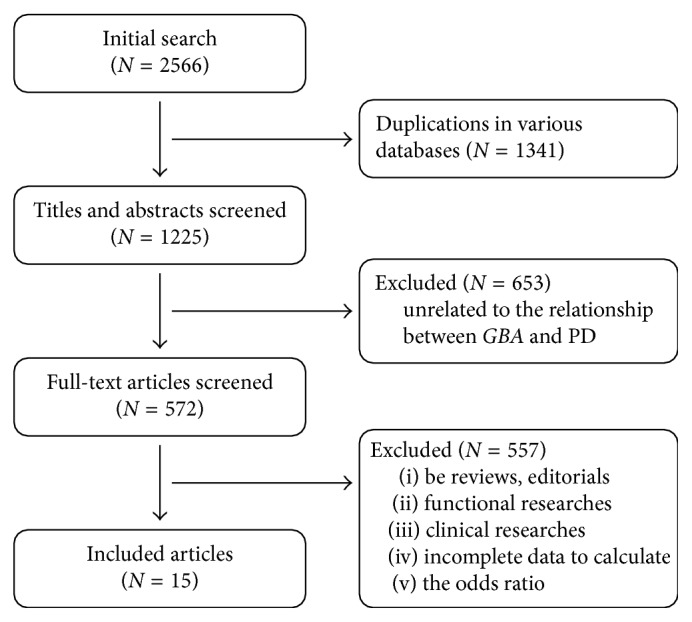
Flowchart of included publications.

According to the standard steps of meta-analysis, a total of 15 publications containing 5,908 PD patients and 5,605 controls were included. The flowchart of screening publications and characteristics of all studies included in the final stages of screening are shown in Figure 1 and Table 1. The NOS scores of each study ranged from 7 to 9, indicating that all of the studies were of good quality.

**Figure 2:**
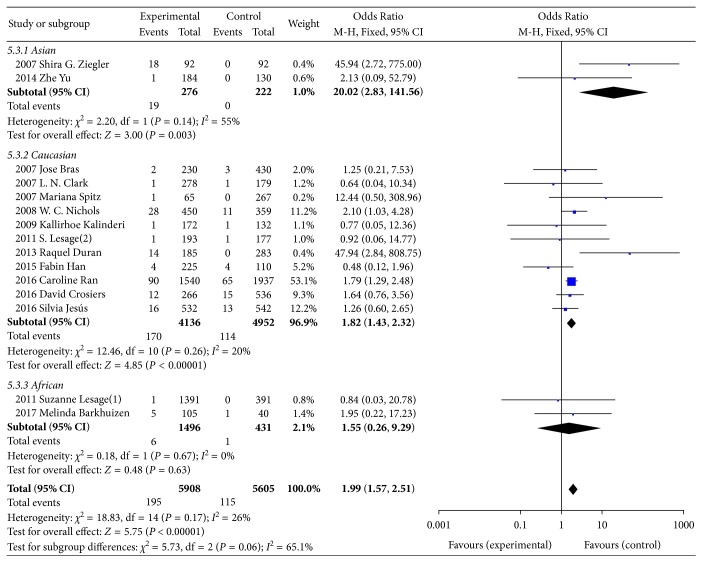
Forest plot of genotype analysis for E326K in PD.

**Figure 3:**
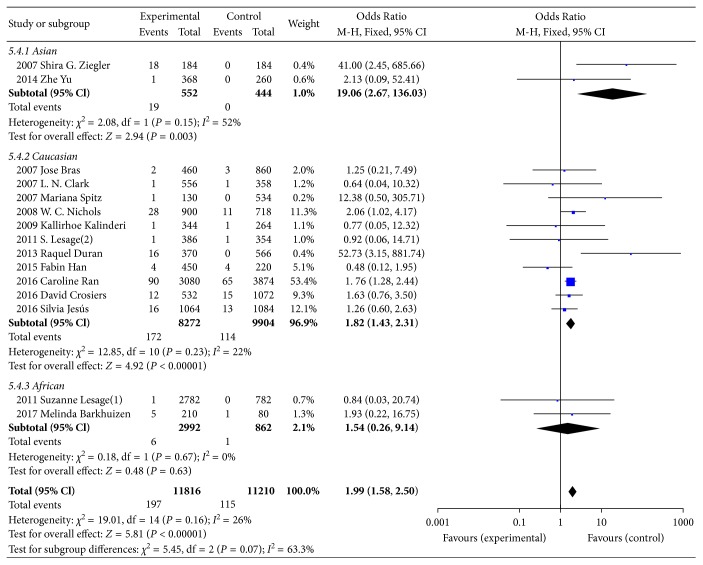
Forest plot of allele analysis for E326K in PD.

In total, there were 195 E326K carriers in the group of PD cases, 2 of which were homozygous, while the other 193 cases were heterozygous. The dominant model (GA+AA/GG) was used to compare the association of E326K and PD. The heterogeneity was acceptable, with a result of *I*^2^ = 26%. Then, the FM was adopted to calculate the genotype association of E326K. The pooled OR of E326K genotype analysis was 1.99, with a 95% CI range from 1.57 to 2.51, as shown in Figure 2, which indicated that E326K is a modest risk factor for PD. In terms of allele frequency comparison between cases and controls, there was no significant difference in heterogeneity (*P*=0.16 and *I*^2^ = 26%). Additionally, the minor allele frequencies of E326K were 1.67% and 1.03%, for PD patients and controls, respectively. The pooled OR for the minor allele A was 1.99, and the 95% CI was 1.58 to 2.50 (Figure 3), which reflects an increased risk of PD. We conducted the subgroup analysis according to Asians, Caucasians, and Africans. We found a significant difference between PD patients and controls for both genotype and allele of E326K in Asians and Caucasians.

**Figure 4:**
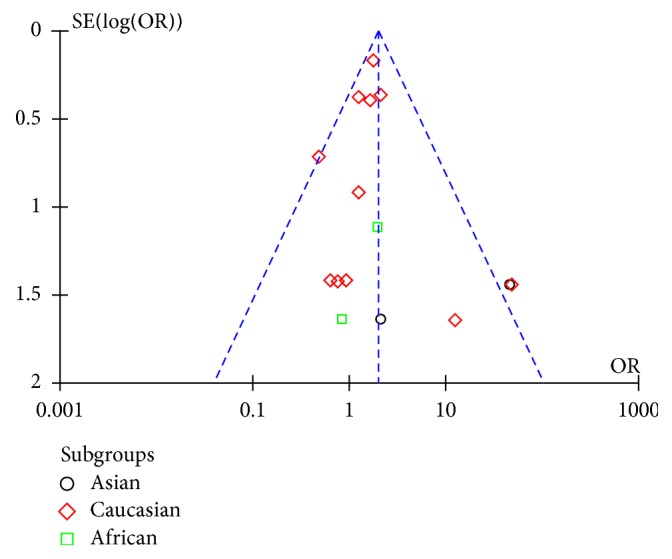
Funnel plot of genotype analysis for E326K in PD.

**Figure 5:**
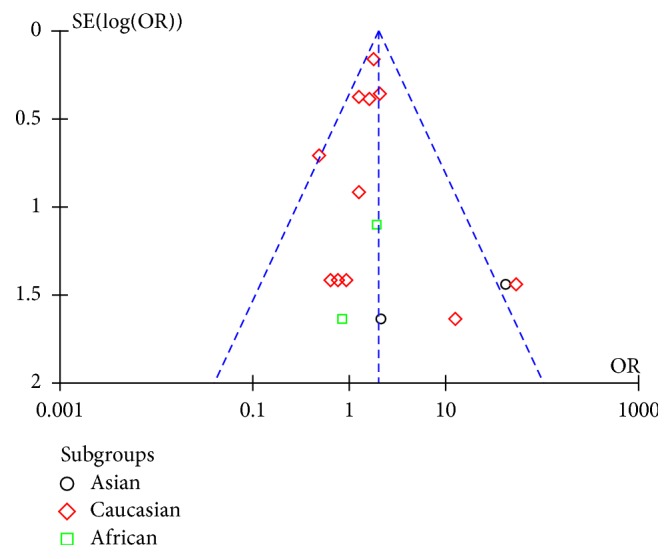
Funnel plot of allele analysis for E326K in PD.

The funnel plots of genotype and allele analyses had a small tendency toward negative results (Figures 4 and 5). When all high quality studies were combined, the pooled ORs were significantly different. By deleting each individual article one at a time, the pooled ORs and 95% CI of each analysis remained stable.

## 4. Discussion

Our meta-analysis demonstrated that a higher proportion of E326K carriers developed PD and the minor allele A was a risk factor for PD. Previously, over 300 variants of *GBA* were reported in PD. However, the replications of those risk variants mostly were not conducted well. For example, after sequencing the a cohort of 519 PD patients and 544 controls, Mitsui et al. [21] found that R120W could increase the risk of PD, which reportedly had no relationship with PD in either Caucasian or Asian [4, 17, 22, 23]. Such cases include H255Q, T369M, D409H, and so on. All these studies were done independently with small sample size, which limited the power to detect the positive relationships between the target variants and PD. Through the method of meta-analysis, we conducted a multicenter, large sample size study. Our study's results were convincing for the following reasons: First, it was conducted with a large, multicenter PD cohort worldwide including 5,908 PD patients and 5,605 controls. Second, all the included publications were of high quality without obvious heterogeneity. Third, even though there was a slight negative reporting bias, the final pooled ORs had positive results. Fourth, the minor allele frequency of E326K among controls in this meta-analysis was 1.03%, which is similar to the allele frequency in the Exome Aggregation Consortium (0.98%).

The *GBA* gene has 11 exons, and the common risk variants N370S and L444P were located near E326K. The OR for E326K was 1.99, which indicates a mild risk for PD when compared with mutations manifesting strong effects, such as N370S and L444P. In a multicenter genetic analysis of *GBA* within the PD population, conducted in 2009 by Sidransky et al. [24], the ORs for N370S and L444P in non-Ashkenazi PD were 3.30 and 9.68, respectively. In Ashkenazi PD patients, the ORs for N370S and L444P were 5.62 and 4.95, respectively. In addition, E326K commonly coexists with other mutations such as N370S and L444P [25]. Moreover, in vitro experiments showed that the GCase activity of both E326K and the L444P variant were lower than that of a single L444P mutation [26]. All of these relationships indicate that E326K has a mild modifying effect on enzyme activity that can participate in the development of PD in a manner similar to other common variants found in genome-wide association studies. Furthermore, in a genetic and phenotypic analysis for a 733 PD patient cohort [27], E326K predicted a more rapid progression of cognitive decline and motor symptom dysfunction, which supports the effect of E326K on the onset of PD.

However, there are some inevitable limitations in this study. First, we did not conduct subgroup analysis according to ethnicity, age at onset, and other related factors due to limited information regarding subgroups. Also, subgroup analysis with an insufficient number of publications may produce a false-positive result. Second, some publications of higher quality were not included due to insufficient data to calculate the ORs and 95% CI. Third, the study did not adjust for age, gender, environment, or other factors.

## 5. Conclusion

In this study, we found that E326K of *GBA* is associated with the risk of PD in total populations, Asians, and Caucasians, respectively. Further studies are needed to clarify the role of *GBA* in the pathogenesis of PD.

## Figures and Tables

**Table 1:** The characteristics of all included publications.

First author, year	NOS	Genetic method	Country	Total number (*N*^a^)		Genotype (GG/GA/AA)	
				Cases	Controls	Cases	Controls
Bras, 2009 [10]	9	PCR and Sanger sequencing	Portugal	230	430	228/2/0	427/3/0
Clark, 2007 [11]	9	PCR and Sanger sequencing	America	278	179	277/1/0	178/1/0
Spitz, 2008 [12]	8	RFLP	Brazil	65	267	64/1/0	267/0/0
Ziegler, 2007 [6]	8	PCR and Sanger sequencing	China	92	92	74/18/0	92/0/0
Nichols, 2009 [13]	8	PCR and TaqMan allelic-discrimination assays	North America	450	359	422/28/0	348/11/0
Kalinderi, 2009 [14]	7	NA	Greece	172	132	171/1/0	131/1/0
Lesage, 2011 [4]	7	PCR and Sanger sequencing	France	1391	391	1390/1/0	391/0/0
Lesage, 2011 [15]	7	NA	North Africa	194 (193)	177	192/1/0	176/1/0
Duran, 2013 [7]	7	PCR and Sanger sequencing	UK	185	283	171/12/2	283/0/0
Yu, 2015 [16]	8	PCR and Sanger sequencing	China	184	130	183/1/0	130/0/0
Han, 2016 [17]	8	PCR and Sanger sequencing	Canada	225	110	221/4/0	106/4/0
Ran, 2016 [8]	8	Pyrosequencing	Sweden	1625 (1540)	2025 (1937)	1450/90/0	1872/65/0
Crosiers, 2016 [18]	8	PCR and Sanger sequencing	Flanders-Belgian	266	536	254/12/0	521/15/0
Jesús, 2016 [19]	8	HRM and direct resequening	Spain	532	542	516/16/0	529/13/0
Barkhuizen, 2017 [20]	8	PCR and Sanger sequencing	South Africa	105	40	100/5/0	39/1/0

NOS: Newcastle–Ottawa Scale; NA: not available; PD: Parkinson's disease; ^a^number of patients whose sequencing results for E326K were available.
